# Motivations of the participants who post a message in an online health forum: a qualitative and quantitative descriptive study in French health forum *Doctissimo*

**DOI:** 10.1186/s12875-022-01906-5

**Published:** 2022-11-29

**Authors:** C. Blanc-Bisson, A.-L. Benazeth, V. Montané, C. Adam, P. Dzeraviashka, Y.-M. Vincent

**Affiliations:** Département de Médecine Générale, Université de Bordeaux, Collège Sciences de La Santé, 146 Rue Léo Saignat, 33076 Bordeaux Cedex, France

**Keywords:** Online health forums, Patient motivations

## Abstract

**Background:**

For more than 20 years, and despite the development of new social networks, health forums have remained a privileged place for people to discuss health issues. This study investigates the motivations of participants to post a message on a French online health forum (called 'Doctissimo') (Forum Santé - Doctissimo,  2022).

**Method:**

Between January 1, 2017 and December 31, 2019, all the first messages recorded on the health forum doctissimo (www.forum.doctissimo.fr) were selected in their initial format by a crawler. The transcripts were imported into the qualitative analysis software Nvivo. Two researchers coded the data until a theoretical saturation was obtained.

**Results:**

We identified four categories of motivation: 1) 'Questioning' allows the exchange of mainly medical information, or sharing of feedback on experiences with the disease: 1722 codes, 44.8%, 2) Worry, need for reassurance: 1066 codes, 27.7% about symptoms or anticipatory anxiety, 3) 'Expressing oneself' mainly allows a catharsis and thus an emotional release, especially negative, but also to share a personal experience: 764 codes 19.9%, 4) Community spirit is a central element to create an emotional support group for psychological support, exchange ideas, meet people in similar situations: 291 codes, 7.6%. The relationship with a health professional when mentioned is generally marked by doubts 39.5%, confusion, or lack of information and the need for additional elements 64.6% or reassurance 60%. The relationship and the obstacles to a medical consultation are described in relation to the use of the forum: immediate availability, anonymity, absence of taboo and community spirit.

**Conclusion:**

The use of information sources offered by the Internet is a way to ask questions, to be reassured, to express oneself or to be confirmed by the community in the hypothesis emitted by a health professional. Patients are looking for an immediate answer, they come to the community for reassurance, they feel free and legitimate.

## Background

The General Practitioner (GP) is the basis of the healthcare system in France [1]. He is himself a powerful therapeutic agent [2]. The social and psychological elements of the relationship with the health professional are of great importance to the patient [3].

In the studies carried out in primary care, the majority of patients expressed the wish that their GP understood their problem, was a skilled listener, empathetic and could provide clear explanations [3–6].

Nowadays, the practitioner is no longer the only source of information, given the increased development of means of communication and exchange. Resources used in the population to answer medical questions outside of consultations are of interest to GPs.

The place of Internet in the world of health is constantly increasing, access to medical information through this mean is possible continuously, a majority of people have permanent access to Internet on their phone [7]. The quality of this information is heterogeneous and difficult to evaluate [8].

Several studies establish links between patients' health pathways and their use of the internet: the 18-month European survey by Santana showed a rapid increase in internet use before the consultation to prepare for the interview and afterwards to find additional information [9]. Farajallah's study found that about 47% of patients reported consulting information on the internet after a consultation with a GP to better understand what the doctor had told them [10]. Other studies elsewhere in the world have found similar results, such as that of Wong in Australia, and confirm the development of a tendency to consult medical data on the periphery of a consultation, particularly in the young population [11].

Online discussion forums are privileged places of expression for users. The conversation is instantaneous, asynchronous, directly transcripted, the anonymity can be guaranteed, the speech is free [12–14]. Several studies have already shown that the use of health forums allows information to be obtained in a context of significant freedom of expression, such as Simoni's study of the online forums of stroke victims [15].

In health care domain, these online forums have a significant audience. In January 2020, 13,903,000 users visited the health forum ‘Doctissimo’ [16] per month, and 939,000 per day [17] ‘Doctissimo’ is one of the most visited French health forum. Created in 2000 by Medcost and managed by a mainly non-medical team, it has been widely established since the late 2000s (It is the 22nd most visited French website of any category in 2008) [18–20].

It is a public forum where anyone can write and read health user opinions and where medical comments are rare [21].

The forums allow access to medical information but also to experiences of living with the disease, tips. Emotions are therefore mixed with medical details [14, 22]. Users go first to a forum that focuses on a health problem similar to their own [23]. The expressed feelings are often polarized (positive and negative) [22] however this dichotomy is not sufficient for the analysis of a forum [24].

As a health professional, the place of these social health networks is undeniable in our practice. In order to understand the expectations of patients, we decided to study social networks related to health and in particular the Health Forum 'Doctissimo'. Our main objective is to describe the motivations for posting a first message, starting a discussion topic and in what proportion, in order to identify what these patients look for in these online health forum.

## Methods

### Study design, sampling, data collection

This study is a qualitative and quantitative, non-directive, retrospective descriptive study. Our main objective was to identify the main motivations of users to start a discussion on the forum. Our secondary objectives were to classify the different motivations into categories and families of categories and then to carry out a statistical analysis of the distribution of these categories: this forum is widely used by many participants, which allowed us to develop a quantitative strategy to complement the qualitative approach.

### Participants

The main characteristics of the participants are to be able to use internet, to be interested in the subject of the forum: health, be able to go to the doctissimo forum, understand the way a forum works, read and write in French.

How we collected data

The forum is divided into different themes created during its development. All first message/post in a given theme was retained and extracted in chronological order of publication.

The identification and extraction of verbatims was carried out by a commonly used automated computer process called CRAWLER.

### Ethical consideration

This is an observational study conducted after the fact, it does not require the information or consent of the people who may have left the data used in this research work according to French law. The consent of the persons having posted is not required since this forum is public and accessible to all. The data extracted from the forum is publicly available. In accordance with the law of bioethics, this research does not require an approval of the bioethics committee and does not fall under the Jardé law. The forum charter available online specifies the conditions of use of this website [25].

### Data analysis

For the qualitative part, the extracted verbatims were double coded by two different people (A. B. and V.M.) as recommended using NVIVO software. During this part of the work, in case of disagreement between the two coders, a third person (YM.V.) was asked to make a decision. The verbatims are analyzed as they are received, using the grounded theory method [26, 27]. The first phase was the identification of categories from a first round of coding of the verbatims. Then the researchers were able to determine the concepts that emerged that eventually led to the theory with the saturation of the data.

The subjects identified in the qualitative analysis were then used to conduct a quantitative analysis using an excel spreadsheet. The posts being numerous it allowed a quantitative analysis by means of the excel software, from obtained qualitative results. Means and percentages were calculated from the identified numbers.

## Results

Between January 1, 2017 and December 31, 2019, 10 234 posts/users were identified. Seven posts were removed from the analysis because of their uninterpretable character or because they were messages from the moderation. The data saturation occurred after the treatment of 1584 posts for qualitative analysis, identifying 3843 codes. Code number could be most important than post number because one post could lead to several codes.

### Request, worry/ need for reassurance, express and community

**Fig. 1:**
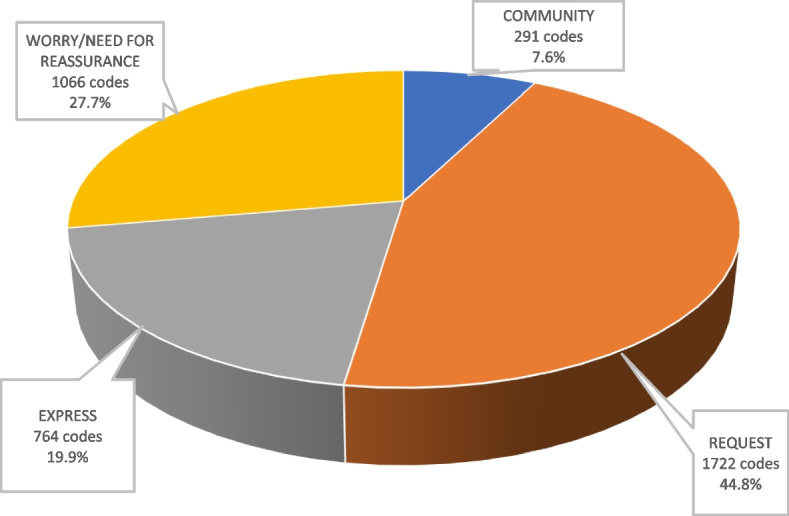
Main users’ motivations on the « Doctissimo» health forum

Four main motivations were identified: questioning in 1722 codes, or 44.8%, was the most frequent subject mentioned, followed by concern and the need for reassurance in 1066 codes, or 27.7%. The need to express oneself was also a feeling expressed in 764 codes, i.e. 19.9%, and finally a community spirit emerged in 291 codes, i.e. 7.6% (Fig. 1).

1. **Medical information and feedback are the main subjects in Request category**

Questioning is the most frequently represented code on the forum (1722 codes, 44.8%). Additional medical information is predominant with 1111 codes, i.e. 64.5% of the questions, different aspects are concerned: the medico-technical side is often addressed, 435 codes, the search for solutions and advice, conduct to follow (317 codes) is very often mentioned as well as for the diagnosis (267 codes) as can be seen in the following verbatim "But during a RMI we cannot diagnose a possible Alzheimer?", "Is it the good finger? […] Is there anything to do? While advice about treatments is less often mentioned (92 codes): "I think I'm running out of treatment […] how can I substitute?" (Table 1).

**Table 1:** Description of the main topics discussed in the theme request. Numbers and frequency in the category and among all codes

REQUEST (*N* = 1722)	Number of codes, N	Percentage in the category, %	Percentage among all codes, %
Medical information	1111	64.5	28.9
Including			
* technical medical question*	435	25.3	11.3
* conduct, advice, solution*	317	18.4	8.2
* diagnosis*	267	15.5	6.9
* advice on treatment*	92	5.3	2.4
Feedback, look for similar experience	453	26.3	11.8
Non medical advice or information	133	7.7	3.5
Deontological/moral/medicolegal ethical advice	25	1.4	0.7

The request to share similar experience or feedback is another theme mentioned on this forum in 453 codes or 26.3% but less frequently found. "Here, I think I have described my symptoms as best I can. Although I do not expect a medical diagnosis from you, you have an experience that no doctor has."

The search for non-medical information or advice was only addressed in 133 codes, i.e. 7.7%. Ethical or deontological aspects are not often highlighted in 25 codes (1.4%).

2. **People look for deviation from standard and anticipation in the category Worry, Need for reassurance**

Worry and the need for reassurance was the second most frequently discussed topic on the forum (1066 codes, 27.7%). For more than half, they concerned a deviation from the situations usually found (522 codes,49.0%), they could be related to symptoms: pain, physical or functional signs (386 codes, 36.2%): "I expose you a problem that worries me a lot […] For the past month I have had a slight pain in my foot.", "[…] What do you think about the kind of blue "patch" or "lump" I have in my knee? […] I noticed it 2–3 weeks ago and it has been worrying me ever since.", "here I am, confusing one word with another trying to find my words,. […] this really worries me». But they could also concern biological results (67 codes, 6.3%), a physical or psychological abnormality (47 codes, 4.4%) or a feeling of loss of control (22 codes, 2.1%) (Table 2).

**Table 2:** Description of the main topics discussed in the theme worry, need reassurance. Numbers and frequency in the category and among all codes

WORRY, NEED REASSURRANCE (*N* = 1066)	Number of codes, N	Percentage in the category, %	Percentage among all codes, %
Deviation from standard	522	49.0	13.6
Including			
* Symptoms*	*386*	*36.2*	10.0
* Biological signs test results*	*67*	*6.3*	1.7
* Feeling of behavioral or physical abnormality*	*47*	*4.4*	1.2
* Feeling of loss of control*	*22*	*2.1*	0.6
Anticipation, event preparation, risk assessment	398	37.3	10.4
Including			
* Evolutionary process*	*197*	*18.5*	5.1
* Having severe illness*	*103*	*9.7*	2.7
* Surgical intervention, future medical contact, hospitalization*	*59*	*5.5*	1.5
* Life choice, life event*	*39*	*3.7*	1.0
Treatment or substance and side effect	76	7.1	2.0
Diagnosis and therapeutic errancy	70	6.6	1.8

Anxiety, including anticipatory anxiety, preparation and risks (398 codes, 37.3%) were fairly widely identified through questions about the evolutionary process (197 codes, 18.5%), the possibility of being very ill (103 codes, 9.7%). They expressed their anticipatory anxiety, or their fears is also widely represented, for example, the fear of having a severe illness with a critical mind: " I'm afraid of having cancer or pulmonary disease, something like that", or fear of surgery: "I'm going to have surgery on my leg and I'm stressed, it’s stupid…".

Far fewer posts were about the surgery itself, finding medical contacts or hospitalization in context (59 codes, 5.5%), suggesting that people are sufficiently informed about these topics. Life choice and life event was a theme discussed (39 codes, 3.7%).

Treatments and their side effects (76 codes, 7.1%) as well as diagnostic or therapeutic wandering (70 codes, 6.6%) were discussed with the same frequency as surgery or hospitalization. Patients seem knowledgeable enough to post few posts on these issues.

3. **Negative feeling/ catharsis and telling one’s story were the most represented posts in the “Express” category**

Their need to express themselves only came in third place in terms of numbers in the topics discussed on the forum (764 codes, 19.9%). It mainly concerned their negative feelings (354 codes, 46.3%) in front of the need to tell their story (281 codes, 36.8%), the positive feelings were much less expressed (103 codes, 13.5%). The need to confide in somebody, to talk is more frequent when the feeling is not good. The ethical or political aspect was rarely addressed in the discussions (Table 3).

**Table 3:** Description of the main topics discussed in the theme Express. Numbers and frequency in the category and among all codes

**EXPRESS (*N* = 764)**	Number of codes, N	Percentage in the category, %	Percentage among all codes, %
Negative feeling, catharsis	354	46.3	9.2
Including			
* Moral suffering/feeling of despair*	*162*	*21.2*	4.2
* Feeling of deadlock/diagnosis impasse*	*62*	*8.1*	1.6
* Feeling of being misunderstood*	*30*	*3.9*	0.8
* Physical suffering/pain*	*22*	*2.9*	0.6
* Feeling of handicap*	*17*	*2.2*	0.4
* Feeling of guilt*	*14*	*1.8*	0.4
* Anger*	*13*	*1.7*	0.3
* Complex*	*12*	*1.7*	0.3
* Emotional traumaBad news*	*9*	*1.2*	0.2
Telling one’s story/testifying	281	36.8	7.3
Including			
* Personal story, history of one’s illness*	*131*	*17.2*	3.4
* Experience*	*62*	*8.1*	1.6
* Knowledge, advice, expertise*	*45*	*5.9*	1.2
* Confidence*	*28*	*3.7*	0.7
* Warning the community about a danger*	*15*	*2.0*	0.4
Positive feeling	103	13.5	2.7
Including			
Helping the community	*53*	*6.9*	1.4
Improvement, healing	*19*	*2.5*	0.5
Recommendations for treatment	*16*	*2.1*	0.4
Resolution, decision	*15*	*2.0*	0.4
Political or ethical opinion	*26*	*3.4*	0.7

Among the negative feelings and catharsis themes, the one that came back the most is moral suffering (162 codes, 21.2%), a feeling of impasse (62 codes, 8.1%), the feeling of being misunderstood (30 codes, 3.9 %) while pain, disability, guilt, anger, complexes, emotional shock or bad news were less mentioned.

In the need to testify, to tell one's story (131 codes, 17.2%) and to share one's experience (62 codes, 8.1%) were the main motivations found, ahead of knowledge, advice, expertise, confidence, or even alerting.

The evocation of positive feelings was found through the need to help the community (53 codes, 6.9%), to testify improvement or the cure’s efficiency (19 codes, 2.5%) then came the recommendations in relation to treatments or decisions made less often. The expression of positive feelings is a minor part: *«with willpower, I stopped to drink every day, […] »*

4. **Psychological support and exchange ideas were expressed in the “Community” category**

Community spirit was another important theme highlighted (*N*=291, 7.6%). We found it through the psychological help (103 codes, 35.4%) that could be requested or provided: "[...] On this forum I am just looking for psychological support [...]" (Table 4). The possibility of exchanging ideas or meeting people in the same situation (86 codes, 29.6%) was also a motivation: « Maybe I could meet people in the same mood, it could be reassuring or depressing ». The possibility of discussing an embarrassing subject (42 codes, 14.4%), advertising or knowledge sharing/accumulation were discussed.

**Table 4:** Description of the main topics discussed in the theme Community. Numbers and frequency in the category and among all codes

**COMMUNITY (*N* = 291)**	Number of codes	% in the category	% among all codes
Psychological support	103	35.4	2.7
Idea exchange, similar people encounter	86	29.6	2.2
Dare to tackle a taboo, embarrassing subject	42	14.4	1.1
Business offers, advertising	32	11.0	0.8
Participatory experience, community survey, knowledge approval	28	9.6	0.7

### Posts after a health professional visit

The mention of a health professional is coded 651 times. In 526 codes (80.9%), users have already consulted a doctor, only 190 (about 29.2%) explicitly referred specifically to their general practionner. The rest either plan to consult, don’t want to, are unable to because of doctor’s unavailablity, or ask if it is necessary.

The proportion of messages mentioning the fact of having already consulted a caregiver before posting a message is in 496 codes.

The distribution of codes for users who have already consulted a doctor is as following: most of the time, patients request further medical information (340 codes, 64.6%), or express a need for additional reassurance, despite the consultation (316 codes, 60%). the patient has doubts about the medical opinion given (196 codes, 39.5%), the lack of information (102 codes, 20.6%) and the lack of detail provided (93 codes, 18.8%) or dissatisfaction (82 codes, 16.5%) are frequently expressed complaints (Table 5). *« I went to meet a doctor […] He […] prescribed paracetamol, I didn’t take it, I think that it is quite a strong drug. Even he told me that it was nothing serious, I am very afraid, stressed, […]»*

**Table 5:** Description of the main topics discussed after visit. Numbers and frequency in the category and among all codes

**AFTER VISIT (*N* = 526)**	Number of codes	% in the category	%among all codes
Request further information	340	64.6	8.8
Need additional reassurance	316	60.0	8.2
Doubt of medical opinion	196	39.5	5.1
Lack of information/confusion	102	20.6	2.7
Without details	93	18.8	2.4
Unsatisfied	82	16.5	2.1
Expressly satisfied	19	3.8	0.5
Things hided	4	0.8	0.1

However, there are 472 codes with a view to reassurance, when the user does not say that they have consulted a health professional (59.9% of codes expressing worry). About 59.5% of the messages did not explicitly mention a relationship with a health care provider in their first message, so it would seem that they would consider solving their problem via the health forum or talking about their situation to the forum community first.

### Immediately available, anonymous, no taboo, a community: the benefits of a forum compared to a consultation

The fact of not having consulted is explained by the immediate of doctors’ unavailability (temporal or geographical), (3.4% of posts). On the forum, there are no spatial, nor temporal barriers which allows the user to ask their question immediately, without having to move: *« I am waiting my doctor for my appointment».*

Categorical refusal to consult is rare (1.6% of the total corpus). Fear or shame of consulting constitute the majority of the refusals.

The notion of community is central, it is possible to talk anonymously to a group. This is the main motivation for about 7.6% of the corpus: *« opening this discussion, I need to share my experience […]. I want us to share our journey ….that ended up negative or positive […], we need to share…»* The forum is used as a place of psychological support, of exchange between fellow human beings which allows to create interpersonal links via Internet, or to approach a taboo subject online because of its embarrassing character: *« I know it's crazy but I don't dare go to my doctor, […}.»*

## Discussion

### Main findings

The obtained results suggest that the main motivations of the users of an online forum are the search for medical information, the request for feedback in order to have the testimony of someone who has experienced the same situation (44.8%), and the need for reassurance in several areas (27.7%). They need to express themselves (19.9%) and feel like they belong to a community (7.6%).

The people who participate in this health forum seem to want to find an answer to a purely medical question, asked anonymously, immediately, within a group. This creates an organizational ease and the possibility to have a quick answer without waiting for an appointment with one's doctor.

The search for feedback from peer is very interesting, because this notion does not exist during a medical consultation, how patients feel and testify is what remains important.

The need for reassurance is also represented, it echoes a concern of the person who posts. This anxiety can be caused by different everyday phenomena. In the majority of cases, it is the occurrence of symptoms (a physically visible sign, a felt functional sign, a pain).

The main purposes that emerge from this work seem to be modelled on the functioning of human-to-human communication. Messages can be used to communicate an emotion or an attitude, to transmit information or to refer to the context, to play with language, to establish a contact with the other, to use language to try to make the recipient (re)act [28].

On this forum, negative effects are over-represented. Indeed, the people who come to express are generally doing it to free themselves from these emotions. Sharing their affects and their mostly negative experiences on the forums allows them to obtain social support from the community [28].

The formulation of their concerns also makes it possible to obtain this support, associated with a reassurance.

The relationships with a health professional reported in our study are often conflicting or express a lack of confidence in the opinion of the medical interlocutor. These doubts seem to be largely explained by a lack of information, and the participants decide to obtain confirmation of the medical opinion on the forum. The therapeutic value of a quality doctor-patient relationship is inestimable [29]. This need for clear information, and therefore understanding of their health problem, seems to be a central concern [30].

This is the main contribution of our study for practitioners: the need to understand the patients' need for support and understanding or else they will not meet their needs and will favour the use of health forums as a substitute for the patient-centred approach.

The patient-doctor relationship should be based on appropriate communication. This communication, if well conducted, should allow for a better analysis of the patient's expectations, whether they are clearly visible or hidden. In this way, emotional needs can be better met and the patient's feelings can be soothed. Creating a space of trust in which the patient feels free to confide is also a challenge in primary care.

### Comparison with other studies

Battaia's work on health discussion forums supports the external consistency of our study. Pure medical information is at the center of concerns [31] for health forums, moreover patients seem to be looking for disease-related life stories which triggers emotions [31]. These do not come into play in the exchange of medical information but only in the exchange of testimonies and feelings about the disease [31]. Rather than of the legitimacy of the professionals it is the experience of living with the disease that is valued [14]. And yet, the study shows that medical information is a central element of the Internet users' stories, and participants need to deepen their knowledge while being reassured [14].

### Strengths and limitations

This study is original: according to our research, there are no similar studies looking at motivations in health forums and primary care. Non-directive, it uses data from a health forum, where expression is free, avoiding a bias in data collection. Only one website was used, it is one of the largest French-speaking health forum [20, the quantity of accessible and collected data is sufficient to carry out a such research. The use of a double coding allows to reduce the bias of subjectivity [32].

Qualitative research is particularly adapted to this work [33], the factors we observe are feelings, designs, emotions, reactions,… we sought to analyze the verbatim and to identify as broadly as possible the topics discussed. The use of a mixed method combining qualitative and quantitative analysis allows to enrich the understanding of the studied phenomenon [32, 33].

Grounded theory analysis is a recognized method in qualitative research [26, 27, 34].

Here, we used raw data from an online health forum, which is a credible source in the field of qualitative research [12]. The analysis made it possible to inductively generate a theorization of the phenomenon of these forums, by proceeding with a conceptualization, a progressive linking of qualitative data [26].

These elements of the study strengthen the internal validity of our research.

However, this work has some limitations. The descriptive and retrospective nature of the study lowers the level of evidence. The representativeness of our sample is difficult to establish because of the fact that only active users have a voice and because of the anonymity of the participants, even if some of them communicate information about themselves, they are few. It would also seem that the audience of this forum is largely female [35]. The forum is strictly French-speaking and therefore accessible only to people who understand that language. This may weaken the external validity of this study.

Silent readers who do not post a first message or participants who have written following a thread are not taken into account in the study, which may create a lack of data, nevertheless the theoretical saturation of the data had been reached following the reading of the 1584 posts entered in the analysis.

## Conclusion

The place of the internet and social networks in our lives is undeniable, and medical practice is no exception. Understanding what patients are seeking and finding online is essential for today's and tomorrow's doctors. This study aims to analyse the verbatims of a French medical forum for the general public to identify what the participants are looking for.

A further quantitative statistical analysis of the database should be led so as to specifically examine the links between 'mentioning a doctor' (before or after a consultation) and the patient’s motivations, and then compare the results with the logique d’information et communaut participants who do not mention a doctor.

## Data Availability

The data used are publicly available and dataset used and analysed during the current study are available from the corresponding author on reasonable request.

## Abbreviation

GP: General Practitionner
